# The prognostic effect of tumor-associated macrophages in stage I-III colorectal cancer depends on T cell infiltration

**DOI:** 10.1007/s13402-024-00926-w

**Published:** 2024-02-26

**Authors:** Umair Majid, Christian Holst Bergsland, Anita Sveen, Jarle Bruun, Ina Andrassy Eilertsen, Espen S. Bækkevold, Arild Nesbakken, Sheraz Yaqub, Frode L. Jahnsen, Ragnhild A. Lothe

**Affiliations:** 1 Department of Molecular Oncology, Institute for Cancer Research, Oslo University Hospital, Oslo, Norway; 2 Institute of Clinical Medicine, University of Oslo, Oslo, Norway; 3 Department of Pathology, Oslo University Hospital-Rikshospitalet, Oslo, Norway; 4 Institute of Oral Biology, University of Oslo, Oslo, Norway; 5 Department of Hepatobiliary Surgery, Oslo University Hospital, Oslo, Norway

**Keywords:** Colorectal cancer, Multiplex fluorescence immunohistochemistry, Tumor associated macrophages, T cells, Immune tumor microenvironment, Prognostic markers

## Abstract

**Background:**

Tumor-associated macrophages (TAMs) are associated with unfavorable patient prognosis in many cancer types. However, TAMs are a heterogeneous cell population and subsets have been shown to activate tumor-infiltrating T cells and confer a good patient prognosis. Data on the prognostic value of TAMs in colorectal cancer are conflicting. We investigated the prognostic effect of TAMs in relation to tumor-infiltrating T cells in colorectal cancers.

**Methods:**

The TAM markers CD68 and CD163 were analyzed by multiplex fluorescence immunohistochemistry and digital image analysis on tissue microarrays of 1720 primary colorectal cancers. TAM density in the tumor stroma was scored in relation to T cell density (stromal CD3^+^ and epithelial CD8^+^ cells) and analyzed in Cox proportional hazards models of 5-year relapse-free survival. Multivariable survival models included clinicopathological factors, MSI status and *BRAF*^V600E^ mutation status.

**Results:**

High TAM density was associated with a favorable 5-year relapse-free survival in a multivariable model of patients with stage I–III tumors (*p* = 0.004, hazard ratio 0.94, 95% confidence interval 0.90–0.98). However, the prognostic effect was dependent on tumoral T-cell density. High TAM density was associated with a good prognosis in patients who also had high T-cell levels in their tumors, while high TAM density was associated with poorer prognosis in patients with low T-cell levels (*p*_interaction_ = 0.0006). This prognostic heterogeneity was found for microsatellite stable tumors separately.

**Conclusions:**

This study supported a phenotypic heterogeneity of TAMs in colorectal cancer, and showed that combined tumor immunophenotyping of multiple immune cell types improved the prediction of patient prognosis.

**Supplementary Information:**

The online version contains supplementary material available at 10.1007/s13402-024-00926-w.

## Introduction

The immune tumor microenvironment (iTME) is an integral part of cancer ecology and plays an important role in tumor development [1]. The iTME consists of diverse cell types and differentiation states, and the cellular composition has a strong prognostic effect in many cancer types [2]. Tumor infiltrating T cells, in particular CD8^+^ T cells, are associated with a favorable prognosis [3], whereas tumor-associated macrophages (TAMs) are generally associated with an unfavorable prognosis [3–5].

In healthy tissue, macrophages have the ability to sense and respond to tissue injury by clearing damaged cells, as well as by recruiting and activating T cells that can help restoring tissue integrity [6]. It is therefore a paradox that TAMs have been shown to promote tumor growth and development of metastases. This can occur through mechanisms such as angiogenesis, production of tumor growth factors, and immunosuppression [5, 7, 8]. TAMs may also have a negative impact on the efficacy of diverse cancer therapies, including immune checkpoint inhibitors, radiotherapy and chemotherapy [9–11].

However, TAMs are a heterogeneous cell population, and specific subpopulations can have anti-tumorigenic properties. Macrophages expressing FOLR2 can prime effector CD8^+^ T cells in the tumor stroma and confer a better survival in breast cancer patients [12]. Subsets of TAMs produce the T-cell attracting chemokine CXCL9 and have positive prognostic associations across several cancer types [13–15]. Furthermore, pharmacological activation of TAMs can drive potent anti-tumor immunity together with activated CD8^+^ T cells in mouse models [16, 17]. Close interaction between T cells and TAMs therefore appears to be important for creation of an anti-tumor microenvironment.

Colorectal cancers (CRCs) have a complex and highly diverse iTME. This is partly determined by genomic factors. In particular, tumors with DNA mismatch repair deficiency and microsatellite instability (MSI) have dense immune cell infiltrations. These hypermutations are also associated with a favorable survival among patients with non-metastatic cancers. However, tumor-infiltrating T cells have prognostic associations beyond the MSI phenotype. This has been thoroughly demonstrated with the Immunoscore [18] and other immunohistochemistry-based approaches [19]. It has also been shown that regulatory T cells can counteract the positive prognostic effect of cytotoxic T cells [19], highlighting heterogeneity of the iTME. Data on the prognostic effect of TAMs in CRC are conflicting, with reports of both a favorable and an unfavorable prognostic effect [20–25]. This potentially reflects the relative presence of subpopulations of TAMs with pro-tumorigenic versus anti-tumorigenic properties [5, 7, 12, 13].

The combination of computational and spatial analysis in digital pathology, with a particular focus on immune cell patterns like macrophages and lymphocytes, provides an intricate understanding of the intra-tumoral immune response, and its implication on survival [26, 27]. This can improve the depth and accuracy of diagnosis and prognosis for cancer patients.

We hypothesized that immunophenotyping of CRCs based on markers for both TAMs and tumor-infiltrating T cells would resolve the prognostic effect. To investigate this we used multiplex fluorescence immunohistochemistry and digital image analyses on tissue microarrays (TMAs) of a single-hospital series of 1720 patients.

## Materials and methods

### Patients and tumor tissue microarrays

Formalin-fixed and paraffin-embedded samples from the primary tumors of two independent cohorts of patients from a single hospital that were treated by surgical resection for stage I–IV CRC were collected from the diagnostic biobank at Oslo University Hospital, Norway (n = 1720; Supplemental Table 1). This included 1429 (83%) patients diagnosed with TNM stage I–III CRC (locoregional disease) and 288 (17%) patients diagnosed with stage IV (distant metastatic disease, missing data for 3 patients). Patient treatment and follow-up were according to standard national guidelines. Clinicopathological data were extracted from the patients’ medical records and registered in a uniform database. Follow-up data for cancer relapse and survival was complete for at least 5 years for all patients except two; data from one patient is missing and one is censored at 4.2 years.

TMAs were constructed from a single tissue core of the central tumor area of blocks selected by an expert pathologist for representativeness, as previously described [19]. Norwegian series 1 (NS1) included patients treated between 1993 and 2003 (n = 922) and the TMAs were constructed from 0.6 mm diameter cores. Norwegian series 2 (NS2) included patients treated between 2003 and 2012 (n = 798) and the TMAs consisted of 1.0 mm diameter cores. MSI status, *BRAF*^V600E^ mutations, *KRAS* mutations [28–31] and T cell markers (CD3 and CD8) have previously been scored [19]. There were no major differences in the distributions of clinicopathological factors or molecular markers between the two series (Supplemental Table 1). We have shown that intraepithelial CD8 and stromal CD3 scores on these TMAs provided similar prognostic power to separate reports on the Immunoscore in multivariable models of stage I–III colon cancers [18], although the Immunoscore considers both the central region and the invasive front of each tumor. This supported representativeness of the TMAs for prognostic analyses of the iTME [19].

### Multiplex fluorescence immunohistochemistry

A multi-color multiplex immunohistochemistry stain was performed on 4 μm thick sections of the TMAs. The staining was performed using antibodies against CD163 (clone EPR14643, Abcam, diluted 1:1000) visualized with Opal 520, CD68 (clone KP1, DAKO/Agilent, diluted 1:3000) visualized with Opal 690, a cocktail of antibodies targeting the epithelial cancer cells (E-cadherin [clone 36, BD-biosciences, diluted 1:20,000], cytokeratin C-11 [Abcam, diluted 1:4000], cytokeratin Type I/II [Thermo Fisher Scientific, diluted 1:2000]) visualized with Opal 570, CD206 (Clone E2L9N, Cell Signaling, diluted 1:1200) visualized with Opal 620, and also included incubation with DAPI for staining of cell nuclei prior to mounting. The stains were carried out using a multiplex kit (NEL810001KT) together with Opal 620 (FP1495001KT, both from PerkinElmer/Akoya, Marlborough, MA, USA). The Opal protocol (PerkinElmer/Akoya) was followed with the exception that slide deparaffinization, antigen retrieval, and antibody stripping were all performed in a PT-link module (DAKO/Agilent, Santa Clara, CA, USA). Slide deparaffinization and initial antigen retrieval were performed at the same time by placing the slides in EnVision FLEX Target retrieval solution, Low pH (DAKO/Agilent) preheated to 65 °C, heating for 20 min at 97 °C and cooling back down to 65 °C. Antibody stripping was performed in the PT-link solution with high/low pH buffers from Akoya, as specified in Supplemental Table 2. During these cycles, slides were placed in solutions preheated to 80 °C, heated to 97 °C for 20 min and cooled back down to 80 °C. All reagents used are specified in Supplemental Table 3. The multiplex protocol was established in “test-TMAs” of samples from several tissue and cancer types prior to staining the NS1 and NS2 TMAs, following a previously described process [32]. In short, this included antibody testing and determination of the optimal antibody titer by chromogenic DAB-based immunohistochemistry, verification of staining patterns in single-plex fluorescence-based immunohistochemistry, verification of complete stripping/denaturing of the antibodies between detection rounds, and verification of staining patterns in multiplex fluorescence immunohistochemistry. Assessments of CD68 and CD163 staining provided by the Nordic immunohistochemical Quality Control (NordiQC; available at http://www.nordiqc.org) were a valuable resource during protocol optimization. The KP1 clone is one of the monoclonal antibodies recommended by NordiQC for staining CD68 by immunohistochemistry.

### Digital image analysis

Stained TMAs were multispectrally imaged using the Vectra 3 system (PerkinElmer/Akoya). A single 20 × (0.5 μm/pixel) image was taken for each sample of the NS1 TMAs, and a 2 × 2 image field was captured for each sample of the NS2. Images were spectrally unmixed, including removal of tissue autofluorescence from the Opal fluorophore signal values, and analyzed by tissue and cell segmentation in inForm software v. 2.3.0 (PerkinElmer/Akoya). The tissue segmentation algorithm was trained on manually input ground truth labels for epithelial cancer cell regions, tumor stroma and background (empty regions on the glass slide). During algorithm training we found areas of high erythrocyte density to be (incorrectly) labeled as epithelial cancer regions. Thus, we also included an erythrocyte region which was subsequently combined with the tumor stroma region during downstream marker scoring according to tumor region in R (described below). Only signals from the epithelial antibody cocktail (stained by Opal 570), DAPI and tissue autofluorescence were used for training. Batch-analysis algorithms were optimized based on a subset of the samples for each of the NS1 and NS2 TMAs. For NS1, images from 16 samples were used to make the batch-analysis algorithm. Five ground truth annotations were made for the epithelial cancer region, seven for tumor stroma, six for background and three for high-density erythrocyte regions during tissue segmentation training. For NS2, images from 15 samples were used. Seven ground truth annotations were made for the epithelial cancer region, 14 for stroma, 14 for background and three for high-density erythrocyte regions. Tissue segmentation training was performed with medium pattern scale, and the analyses were output with “fine” segmentation resolution. The minimum segment size was set to 1000 pixels. Both algorithms achieved ≥98% training accuracy based on the ground truth labels. For cell segmentation, the same settings were used for both series/algorithms, and individual nuclei were first segmented with the counterstaining approach based on DAPI signals. Minimum size was set to 80 pixels, with a typical size of 320 pixels. Minimum signal was set to 0.24, splitting was set at 2.26 and growing was set to 0.35. Membrane signal was used to aid segmentation. For cytoplasm segmentation, the inner distance to nucleus was set to 1 pixel, outer distance to 6 pixels and minimum size was set to 20 pixels. For membrane segmentation, Opal 690 full scale count was set to 18, Opal 520 to 10, Opal 570 to 30 and Opal 620 to 30. Distance to membrane (for maximum cell size determination) was set to 12 pixels. All images were manually inspected in the review tab in inForm after batch analysis of the two TMA-cohorts, and tissue folds and necrotic regions were manually marked and excluded from the image analysis. A flow-chart for the digital image analysis and marker scoring pipeline is outlined in Supplemental Fig. 1.

### Marker scoring

Data tables with raw mean fluorescence intensity values per marker per cell for each TMA core were exported from inForm and further processed in R (v. 3.6.3). Each individual cell in each individual TMA core was scored as positive/negative for CD68 and CD163 based on the mean nuclear signal intensity of their corresponding fluorophores in each patient series (normalized counts, total weighting in inForm software; Supplemental Table 4). The nuclear signal was used since marker signals in this cell segment were sufficient to score cells as positive or negative, and nuclear segmentation was more consistent than cytoplasmic and membrane segmentation. The data on CD206 were not included in the current study due to poor technical quality.

**Fig. 1:**
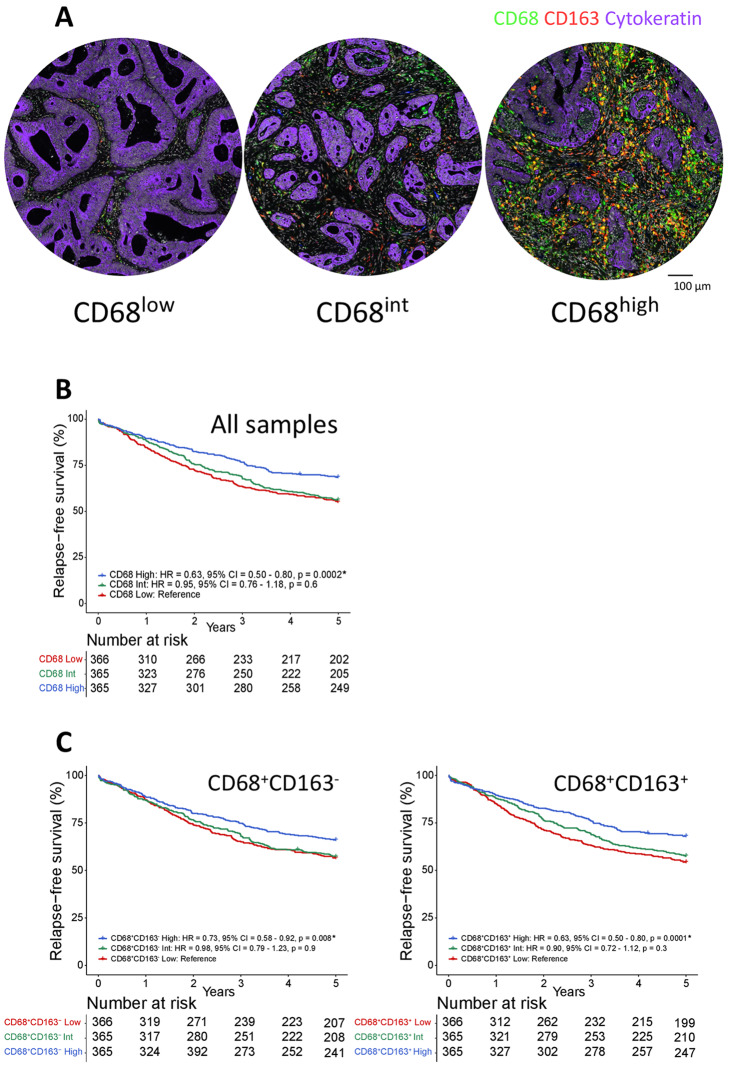
Prognostic effect of TAMs in stage I–III CRC. **A** Three TMA cores stained against CD68 (green), CD163 (red) and cytokeratin (purple) illustrate tumors with low (left), intermediate (middle) and high (right) numbers of CD68^+^ TAMs. **B** Kaplan-Meier plot of 5-year RFS according to the stromal density of CD68^+^ TAMs (high, intermediate, low) among patients treated by complete resection for stage I–III CRC (the two patient cohorts NS1 and NS2 combined; n = 1096). **C** Kaplan-Meier plots for the same analysis as in **B**, but here stratified according to the stromal densities of CD68^+^CD163^-^ (left) and CD68^+^CD163^+^ (right) TAMs. Hazard ratios (HR), *p*-values and 95% confidence intervals (CI) are from Cox proportional hazards analyses and *p*-values. *Significant values *p* < 0.05 (Color figure online)

Tumor-infiltration with macrophages was most prominent in stromal regions, outside of the cytokeratin positive regions marking the tumor epithelium (Fig. 1A). Macrophage infiltration was therefore scored in the stromal tissue compartment of each TMA core and normalized by dividing by the stromal tissue area (in mm^2^) of the individual core.

Tissue cores with poor technical quality upon manual image inspection were excluded from downstream analysis (n = 191). Additional samples (n = 69) were excluded according to the following data quality control criteria: less than 5% malignant epithelial tissue; fewer than 100 malignant epithelial cells; fewer than 50 stromal cells; or a total tissue area of less than 150,000 pixels (0.0375 mm^2^).

### Survival analyses

The study follows the Reporting recommendations for tumor marker prognostic studies (REMARK; Supplemental Table 5). The primary endpoint was 5-year relapse-free survival (RFS), analyzed as the time from surgery to recurrence or death from any cause as defined by Punt et al. [33]. Analyses of RFS were performed in patients with a free resection margin >1 mm and no residual tumor (R0 status) and therefore excluded stage IV cancers. Patients who received pre-operative chemoradiotherapy (n = 32), had synchronous primary CRCs (n = 29), or were registered with an RFS of 0 (n = 4) were also excluded, leaving 1096 patients with stage I–III CRC and good technical quality of immunohistochemistry data for analyses. Survival analysis of patients with stage IV CRC was performed with 5-year overall survival (OS) as the endpoint, since the majority of these patients did not receive radical treatment. Complete 5-year OS data was available for all patients and was calculated from the time of surgery of the primary tumor (n = 248 patients with good technical quality of immunohistochemistry data). Uni- and multivariable Cox proportional hazards models of immune cell infiltration scores were calculated with the *coxph* function in the R package *survival* (v. 3.1-8). Potentially confounding variables included in multivariable analysis of CD68 (Table 1) were pT and pN stage, tumor location, MSI status, *BRAF*^V600E^ mutational status, patient age and sex. Multivariable survival analysis was stratified by cohort (NS1 and NS2). Patients were excluded from multivariable analysis if data was missing for any of the variables included. The assumptions of proportional hazards were tested using the *cox.zph* function in the *survival* package. Kaplan-Meier plots were made using the *survminer* package (v. 0.4.9), while statistics presented in the plots are based on the Cox proportional hazards model, as described above.

**Table 1:** Uni- and multivariable analysis of 5-year RFS according to macrophage density in stage I–III CRC (n = 1013)

	Univariable analysis			Multivariable analysis		
				c-index (concordance): 0.677 (standard error = 0.013)		
Variable^a^	HR	95% CI	*p*-value	HR	95% CI	*p*-value
Stromal CD68 per mm^2^ (log2-transformed)	0.92	0.89–0.96	0.0001	0.94	0.90–0.98	0.004
Sex						
Women vs men	0.95	0.78–1.15	0.6	0.81	0.66–1.00	0.05
pT						
T2 vs T1	0.92	0.52–1.63	0.8	0.89	0.50–1.59	0.7
T3 vs T1	1.76	1.05–2.95	0.03	1.40	0.82–2.39	0.2
T4 vs T1	2.99	1.60–5.58	0.0006	3.01	1.59–5.71	0.0007
pN						
N1 vs N0	1.61	1.28–2.03	<0.0001	1.60	1.26–2.02	<0.0001
N2 vs N0	2.76	2.07–3.67	<0.0001	2.56	1.90–3.43	<0.0001
MSI status						
MSI vs MSS	0.71	0.53–0.93	0.01	0.55	0.37–0.83	0.005
*BRAF* status						
Mutated vs wild-type	1.09	0.84–1.41	0.5	1.60	1.10–2.32	0.01
Location						
Rectum vs left	0.76	0.58–0.99	0.04	0.95	0.72–1.25	0.7
Right vs left	0.85	0.69–1.07	0.2	0.85	0.67–1.09	0.2
Age^b^	1.03	1.02–1.04	<0.0001	1.04	1.03–1.05	<0.0001

^a^Including patients with complete data for all variables (n = 1013)

^b^Violates proportional hazards assumption in univariable analysis. The multivariable analysis was therefore also tested without age as a confounder, but this did not affect the analysis in any significant way, and in particular did not affect the prognostic value of CD68

### Statistics

All statistical analyses were performed in Stata and RStudio v. 1.1.383 with R v. 3.6.3. All statistical tests were two-sided and *p*-values less than 0.05 were considered significant. Group comparisons were performed using Kruskal-Wallis and Wilcoxon tests, while correlations were measured by the Pearson correlation coefficient.

## Results

### High density of tumor-associated macrophages is associated with better prognosis in stage I–III microsatellite stable CRC

The density of TAMs in the stromal compartment of CRCs was evaluated by multiplex fluorescence immunohistochemistry of a pan-TAM marker (CD68) and a marker for a TAM sub-population (CD163; Fig. 1A) on TMAs of primary tumors from 1720 stage I–IV CRCs (collected in two time periods at a single hospital; Supplemental Table 1). Samples (n = 260, 15%) with poor technical quality evaluated upon manual image inspection (large tissue folds or necrotic regions) or that didn’t meet the data quality control parameters (criteria specified in the Materials and Methods section) were excluded, leaving 1460 samples for further analysis. 44% of CD68^+^ TAMs co-expressed CD163 (Supplemental Table 1). The density of TAMs decreased with each progressive cancer stage (Supplemental Fig. 2), and locoregional cancers (stage I–III) had a higher density of CD68^+^ TAMs than cancers with distant metastasis (stage IV) irrespective of CD163 expression (Supplemental Fig. 3).

**Fig. 2:**
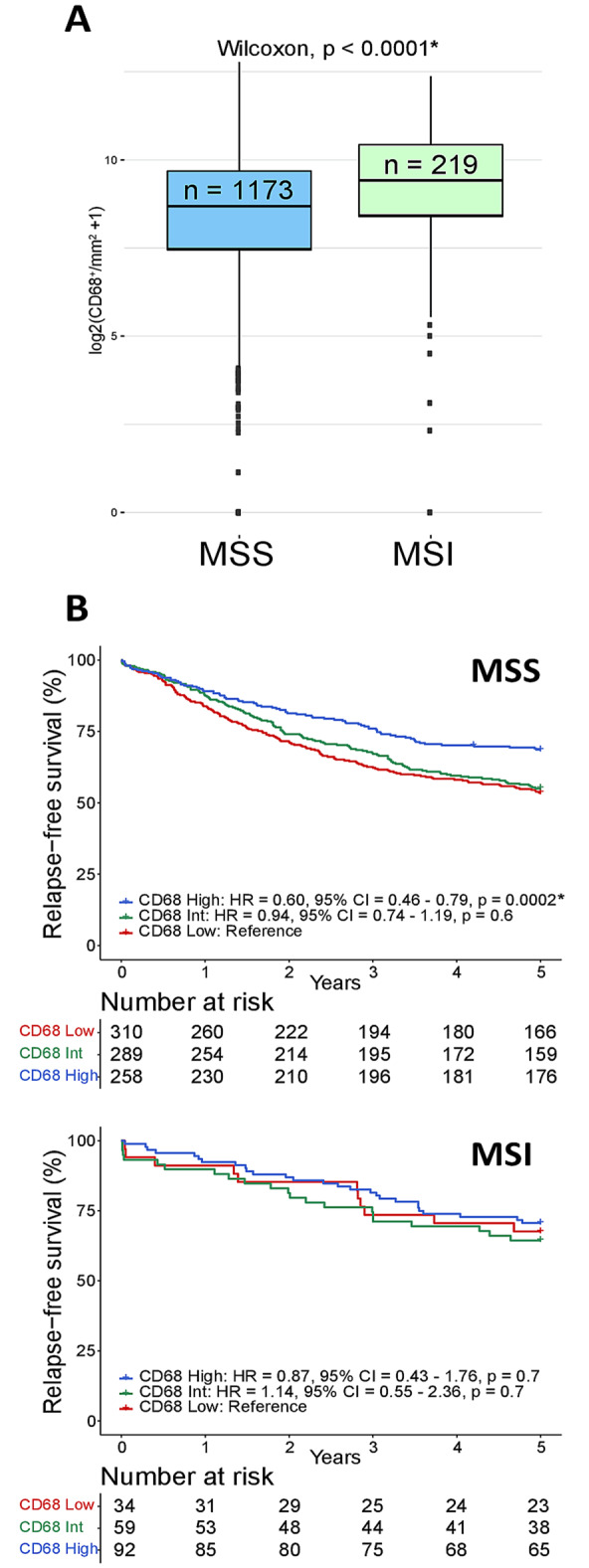
Density and prognostic effect of CD68^+^ TAMs relative to MSI status. **A** Box plots of TAM densities (CD68^+^ per mm^2^, log2 transformed) in primary MSS and MSI tumors of stage I–IV CRCs. *P*-value was estimated by the Wilcoxon test. **B** Kaplan-Meier plots of 5-year RFS according to the stromal density of CD68^+^ TAMs in MSS and MSI tumors separately for patients treated by complete resection of stage I–III CRC. Hazard ratios (HR), *p*-values and 95% confidence intervals (CI) are from Cox proportional hazards analyses and *p*-values. *Significant values *p* < 0.05

Tumors were stratified into three equally sized groups based on the stromal density of CD68. The group with low density had less than 268 CD68^+^ cells per mm^2^ of tumor stroma, the intermediate group had between 268 and 707, and the group with high density had above 707 CD68^+^ cells per mm^2^ of tumor stroma (maximum density was 7010 CD68^+^ cells per mm^2^). Analysis of 5-year RFS in patients treated by complete resection for stage I–III CRC showed that high infiltration of CD68^+^ TAMs was associated with a significantly better survival than lower infiltration, both when examining the two cohorts together and separately (Fig. 1B and Supplemental Fig. 4). This was not dependent on the expression of CD163, since high density of both CD68^+^CD163^−^ and CD68^+^CD163^+^ TAMs were associated with better 5-year RFS than intermediate/low densities (Fig. 1C). Further analyses were therefore performed based on CD68^+^ TAMs. The density of CD68^+^ TAMs was prognostic also in a multivariable analysis with clinicopathological and genetic prognostic markers (Table 1). There was no difference in the prognostic effect of CD68 according to whether patients with stage III cancers were treated with adjuvant chemotherapy or not (*p*_interaction_ = 0.9, with CD68 as continuous variable). However, TAM densities and their prognostic value differed according to tumoral MSI status. MSI tumors had a significantly higher TAM density than microsatellite stable (MSS) tumors (Fig. 2A), and the prognostic value of CD68^high^ was exclusive to patients with MSS tumors, although the interaction test was not significant (*p*_interaction_ = 0.8, with CD68 as continuous variable) (Fig. 2B). There was no difference in the 5-year overall survival of patients with stage IV cancers according to the density of CD68 (Supplemental Fig. 5).

### Prognostic value of TAMs depends on the number of tumor-infiltrating T cells

**Fig. 3:**
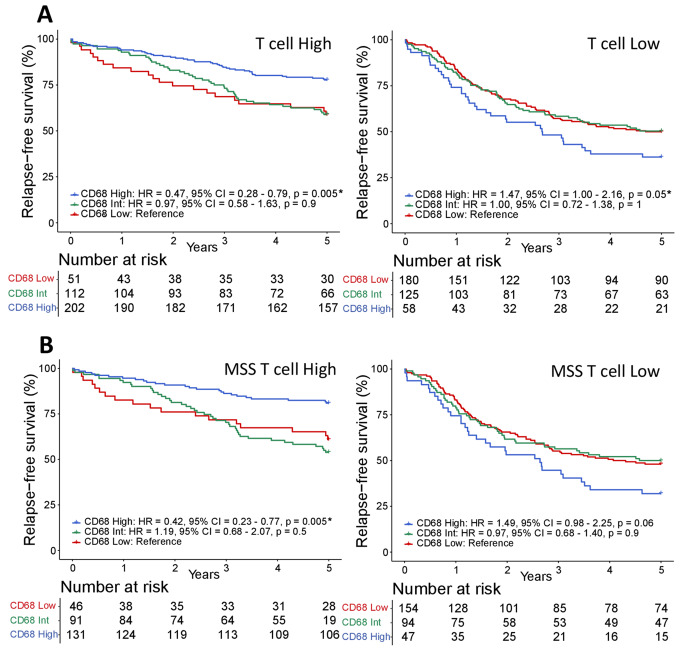
Prognostic heterogeneity of CD68^+^ TAMs relative to tumor-infiltrating T cells. **A** Kaplan-Meier plots of 5-year RFS according to the stromal density of CD68^+^ TAMs in stage I–III CRCs with high (stromal CD3^high^ and epithelial CD8^high^) and low (stromal CD3^low^ and epithelial CD8^low^) T-cell densities separately. **B** Kaplan-Meier plots from the same analysis as in **A**, but including MSS tumors only. Hazard ratios (HR), *p*-values and 95% confidence intervals (CI) are from Cox proportional hazards analyses and *p*-values. *Significant values *p* < 0.05

The density of TAMs correlated with the density of stromal CD3^+^ and epithelial CD8^+^ T cells in CRCs (Pearson’s correlation 0.4 and 0.3, respectively, *p* < 0.0001; Supplemental Fig. 6). We have previously shown that low levels of these T-cell populations are associated with a poor survival in this patient cohort [19]. Here, we performed a stratified analysis of TAM density according to T cells. CD68^high^ was associated with a favorable 5-year RFS among patients with T^high^ tumors (epithelial CD8^high^ and stromal CD3^high^; Fig. 3A). In contrast, CD68^high^ was associated with a poor survival among patients with T^low^ tumors (epithelial CD8^low^ and stromal CD3^low^; p_interaction_ = 0.0006). TAMs had no prognostic effect in patients with mixed levels of the two T-cell populations (stromalCD3^high^ epithelialCD8^low^ and stromalCD3^low^ epithelialCD8^high^; Supplemental Fig. 7). The prognostic heterogeneity of CD68^high^ according to high and low T-cell density was consistent among patients with MSS tumors separately (*p*_interaction_ = 0.0007; Fig. 3B), while a similar analysis of MSI tumors was inconclusive due to low patient numbers in several of the subgroups (Supplemental Fig. 8).

## Discussion

This study shows that integrated immunophenotyping based on TAMs and tumor-infiltrating T cells can specify the prognostic heterogeneity of TAMs in locoregional CRCs. While high TAM density was a positive prognostic factor in general, the prognostic effect was dependent on the presence of T cells, and high TAM densities appeared to confer a particularly poor patient survival when found in tumors with low T-cell densities. This apparent dichotomy may, at least in part, be explained by a bidirectional interaction between TAMs and T cells. An iTME rich in T cells contains activated and IFN-γ producing T cells [34]. Macrophages become highly phagocytic and efficient at antigen presentation in response to IFN-γ [35]. IFN-γ activated TAMs may thus attack and phagocytose malignant cells and provide anti-tumor activity through their capacity to stimulate T cells and produce chemokines that attract T cells [13, 14, 36]. Thus, TAM activation may create a positive feedback loop for further recruitment and activation of T cells. In contrast, in a T-cell deprived iTME, it is likely that pro-tumorigenic TAMs will dominate and promote angiogenesis, produce tumor growth factors and suppress other immune cells [5, 7, 8]. A larger panel of TAM markers to delineate various populations, along with integrated spatial analysis with T-cell populations is needed to confirm this dichotomy.

Multiple studies have investigated the prognostic value of CD68^+^ TAMs in CRC, several of which have reported that high numbers of TAMs are associated with better patient prognosis [7, 37–39]. This is in contrast to most other solid tumor types, and the definite reasons for this disparity are yet to be completely understood. Intestinal macrophages are constantly replenished from blood monocytes and interact with commensal bacteria and the dynamic intestinal niche [40–42]. Intestinal macrophages and TAMs in CRC may therefore have different properties from TAMs in other cancers.

The prognostic effect of TAMs was exclusive to patients with MSS tumors. The lack of a prognostic impact in the MSI setting may be associated with the good survival of these patients in general. However, the favorable prognostic effect of MSI is attributed to a high infiltration of T cells in response to the thousands of neoantigens produced in these mismatch repair deficient tumors. Our study was not sufficiently powered to discern the prognostic heterogeneity of TAMs in relation to both T-cell densities and MSI status. Nonetheless, approximately 85% of all primary CRCs have the MSS phenotype, and our results are representative for the majority of patients with locoregional CRC.

The heterogeneous prognostic effect suggests that TAMs could be important targets for anti-tumor therapy in CRC [36]. Macrophages are extremely plastic cells that differentiate in response to cues from the microenvironment [43]. This plasticity can be exploited therapeutically, and several studies have shown that macrophages can be reprogrammed in vivo [16, 17, 44]. A better understanding of which TAM phenotypes constitute the CD68^high^ populations in the T-cell rich versus T-cell depleted iTME of CRC will be important to identify new therapeutic targets.

A limitation of this study is that TMAs only include a small part of the original tissue sample and offer a restricted view of the iTME in the tumor compared to whole tissue sections. There are immunological differences within a tumor. However, our study demonstrates that the immunological trends within a tumor, represented with a large sample size from central tumor regions, have predictive value. Another limitation is that the macrophage and T-cell markers were analyzed on serial sections. Other techniques for even higher-plex fluorescence immunohistochemistry, like CODEX and BLEACH &STAIN, could be valuable to analyze these and additional markers within the same tissue section, and would allow for even more advanced spatial analyses. These approaches do, however, have drawbacks, such as increased cost, standardization challenges, and the requirement for significant data analysis in addition to their technological complexity. Serial tissue section is a conventional and economical technique with well-established guidelines for regular pathological diagnostic assessment.

Integrated analysis of multiple immune cell markers on tissue sections can be compatible with standard diagnostics, as equipment for simultaneous in situ evaluation of three immune markers is available in many pathology laboratories today. Alternatively, tumor-infiltrating T cells and TAMs can be stained on serial tissue sections, as was performed in this study. There is a need to validate the superior prognostic power and to develop the optimal assay for combined evaluation of T cell and TAM counts in independent cohorts.

## Conclusions

We define prognostic heterogeneity of TAMs in CRC and suggest that combined quantitation of both T cells and TAMs can increase the prognostic power of iTME tests compared to examining T cells alone.

## Electronic supplementary material

Below is the link to the electronic supplementary material.

Supplementary Material 1

Supplementary Material 2

Supplementary Material 3

Supplementary Material 4

Supplementary Material 5

Supplementary Material 6

Supplementary Material 7

Supplementary Material 8

Supplementary Material 9

Supplementary Material 10

## Data Availability

All data generated or analyzed during this study are included in this published article. Immunohistochemistry data of macrophage markers will be made available upon reasonable request to the corresponding author.

## List of abbreviations

CRC: Colorectal cancer
iTME: Immune tumor microenvironment
TAMs: Tumor-associated macrophages
TMA: Tissue microarray
MSI: Microsatellite instable
MSS: Microsatellite stable
RFS: Relapse-free survival
