# Supplementary figures and images for: The prognostic effect of tumor-associated macrophages in stage I-III colorectal cancer depends on T cell infiltration
Source: Cell Oncol (Dordr). 2024 Feb 26;47(4):1267–76. doi: 10.1007/s13402-024-00926-w (PMC11322253; doi:10.1007/s13402-024-00926-w)

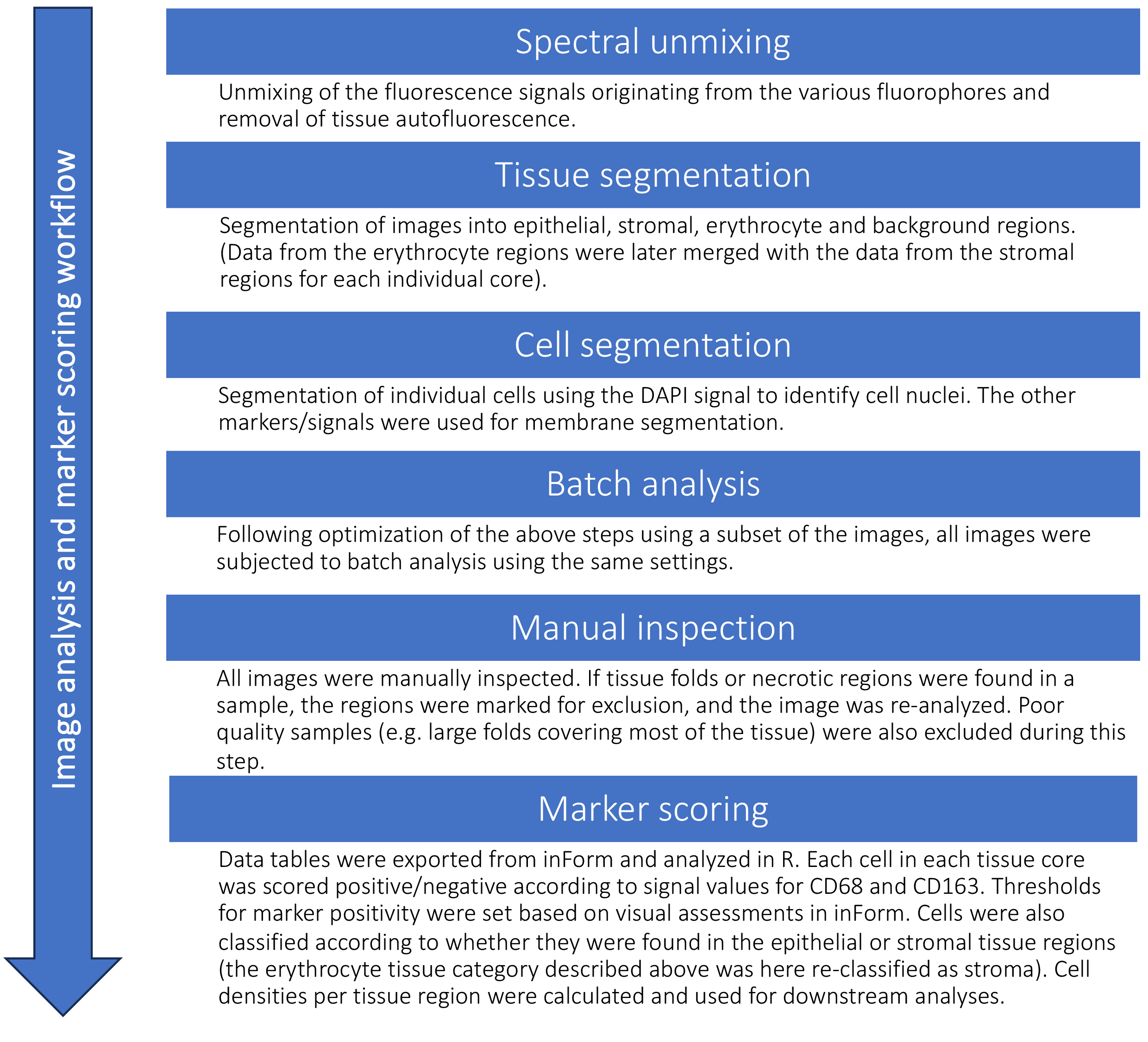

Supplement: Supplementary file 1 — Supplementary Material 1 [file 13402_2024_926_MOESM1_ESM.tiff]

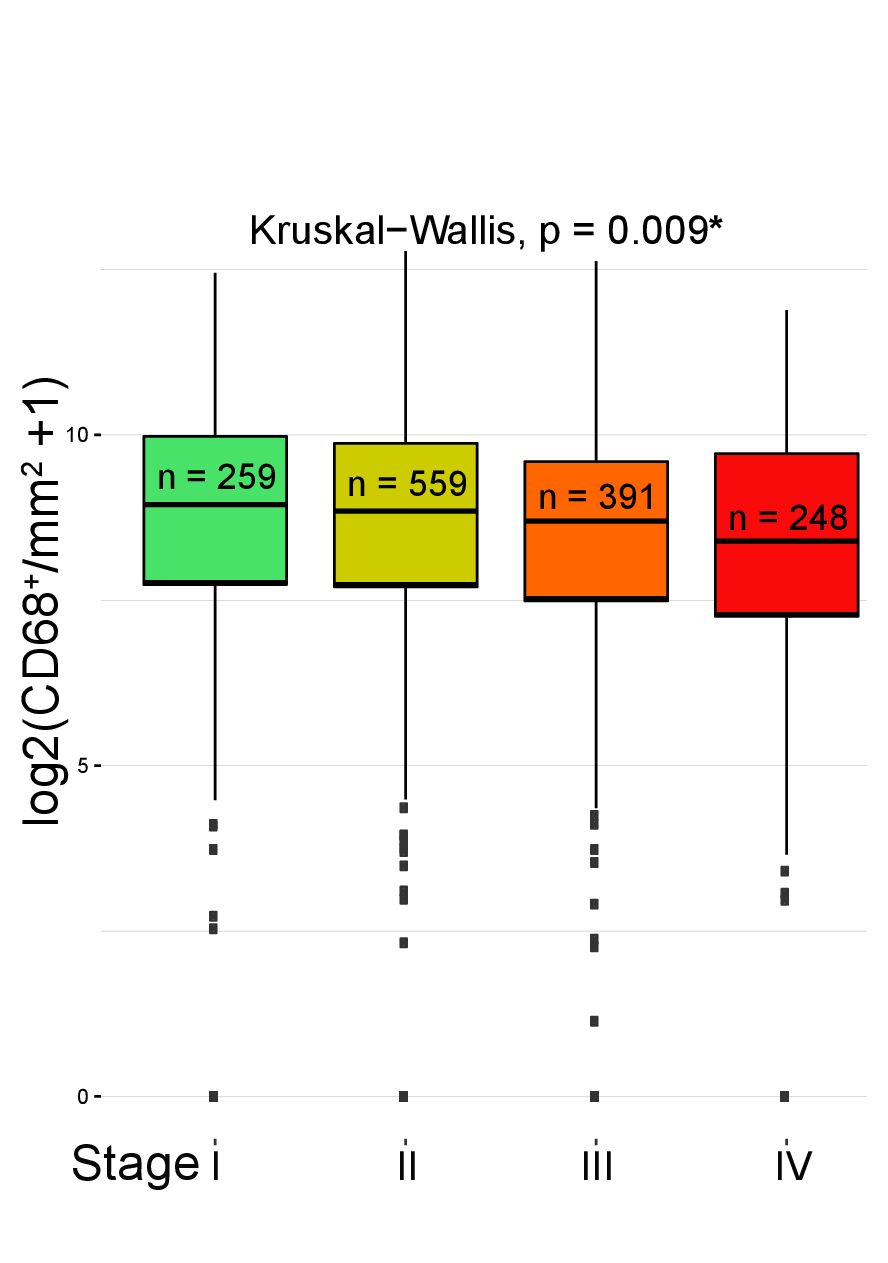

Supplement: Supplementary file 2 — Supplementary Material 2 [file 13402_2024_926_MOESM2_ESM.tiff]

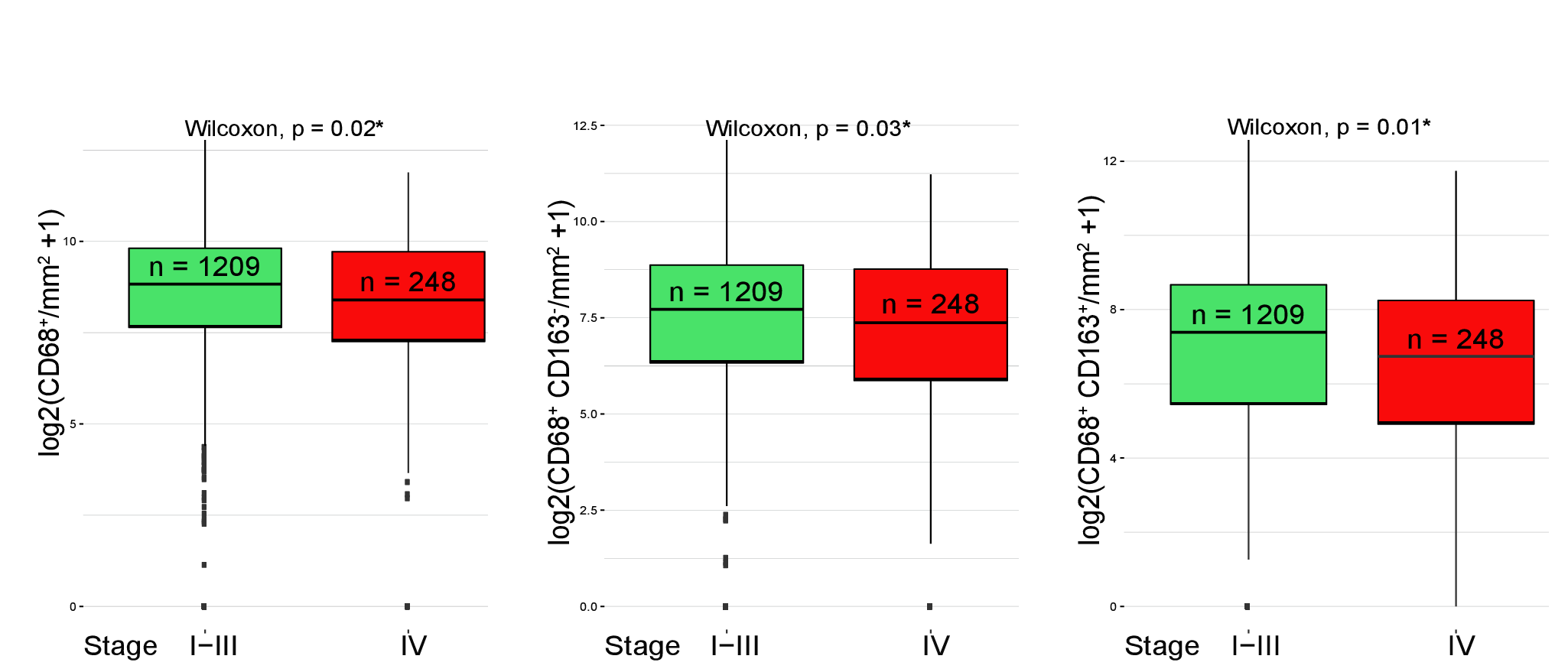

Supplement: Supplementary file 3 — Supplementary Material 3 [file 13402_2024_926_MOESM3_ESM.tiff]

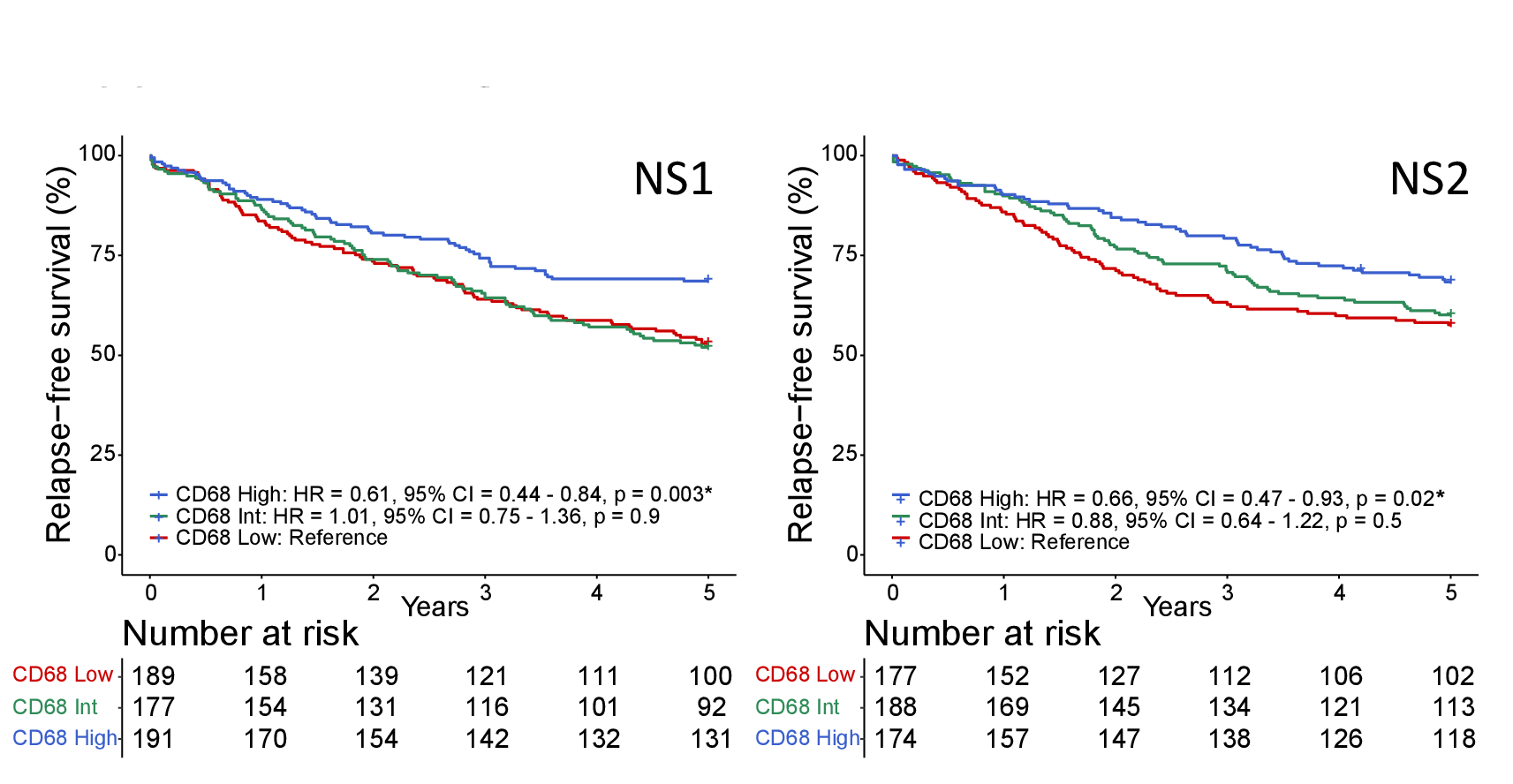

Supplement: Supplementary file 4 — Supplementary Material 4 [file 13402_2024_926_MOESM4_ESM.tiff]

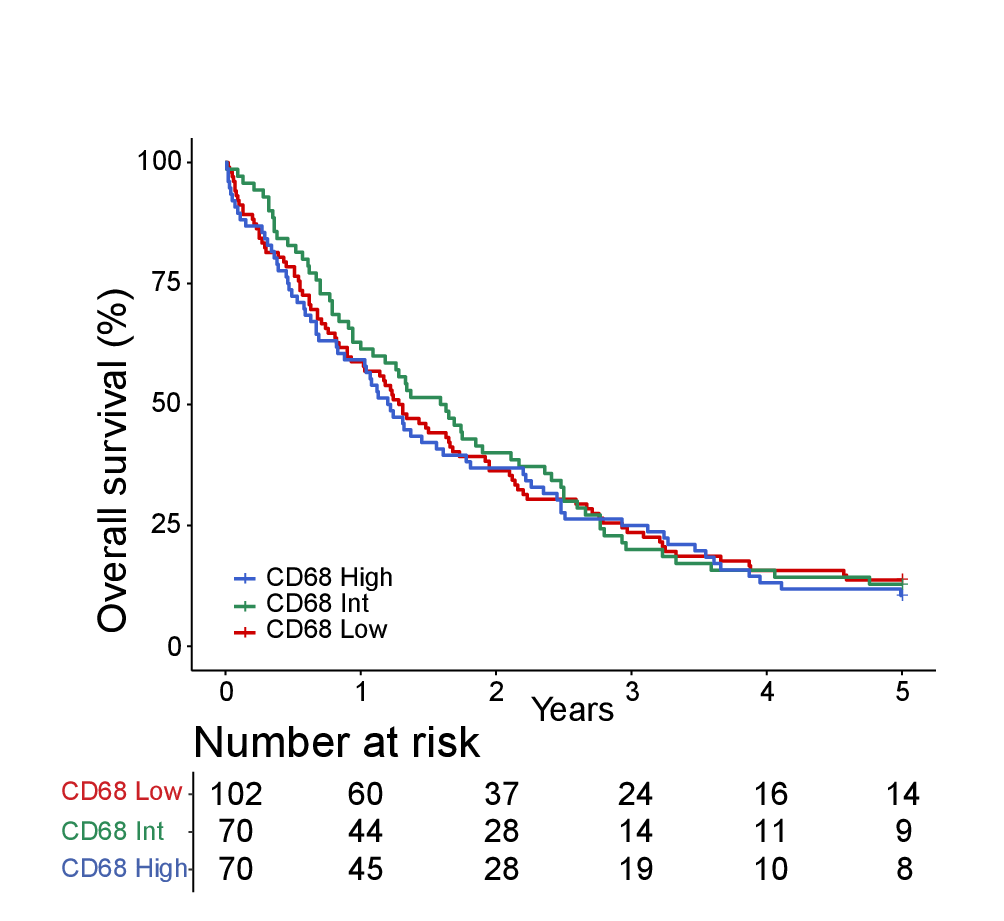

Supplement: Supplementary file 5 — Supplementary Material 5 [file 13402_2024_926_MOESM5_ESM.tiff]

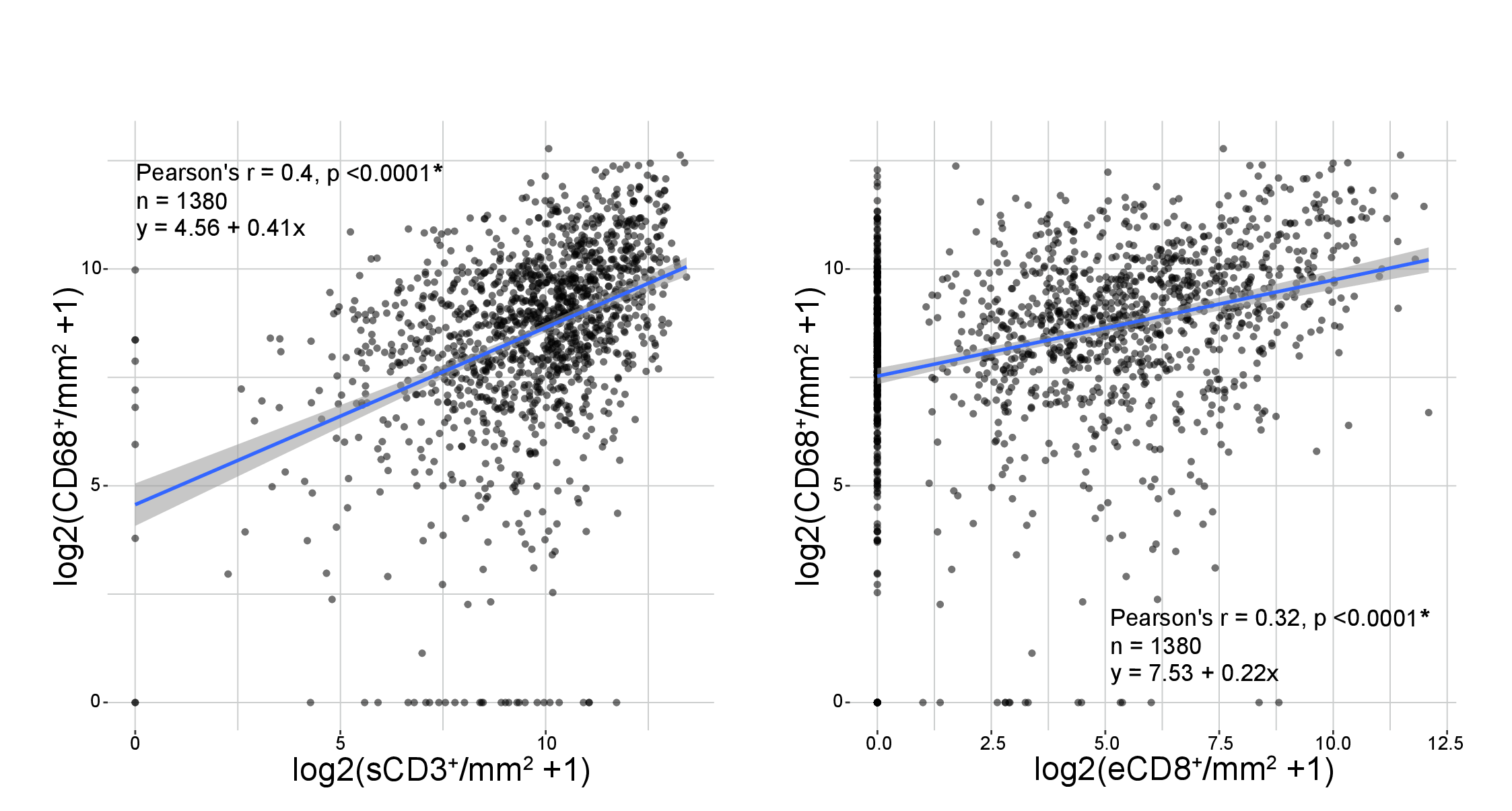

Supplement: Supplementary file 6 — Supplementary Material 6 [file 13402_2024_926_MOESM6_ESM.tiff]

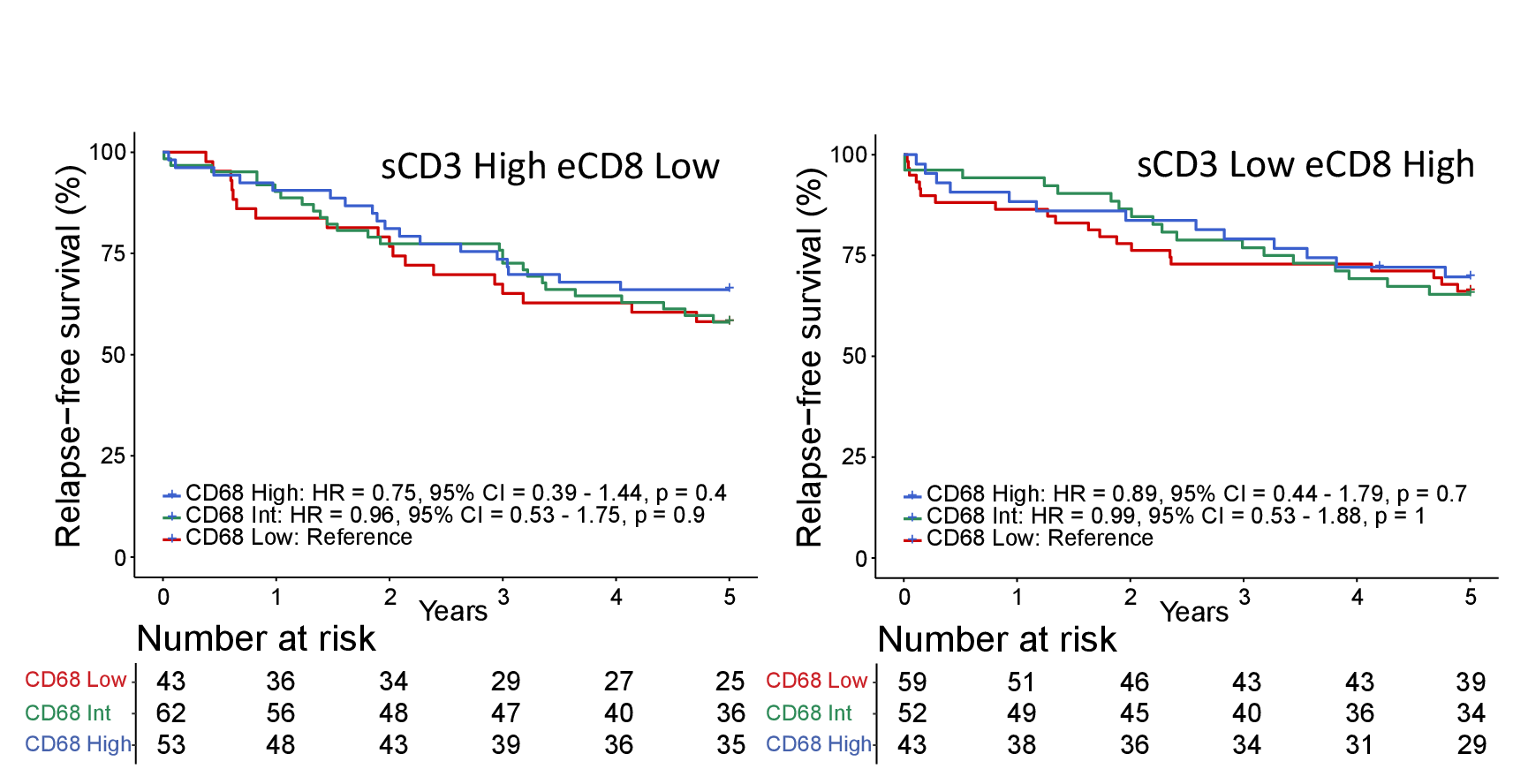

Supplement: Supplementary file 7 — Supplementary Material 7 [file 13402_2024_926_MOESM7_ESM.tiff]

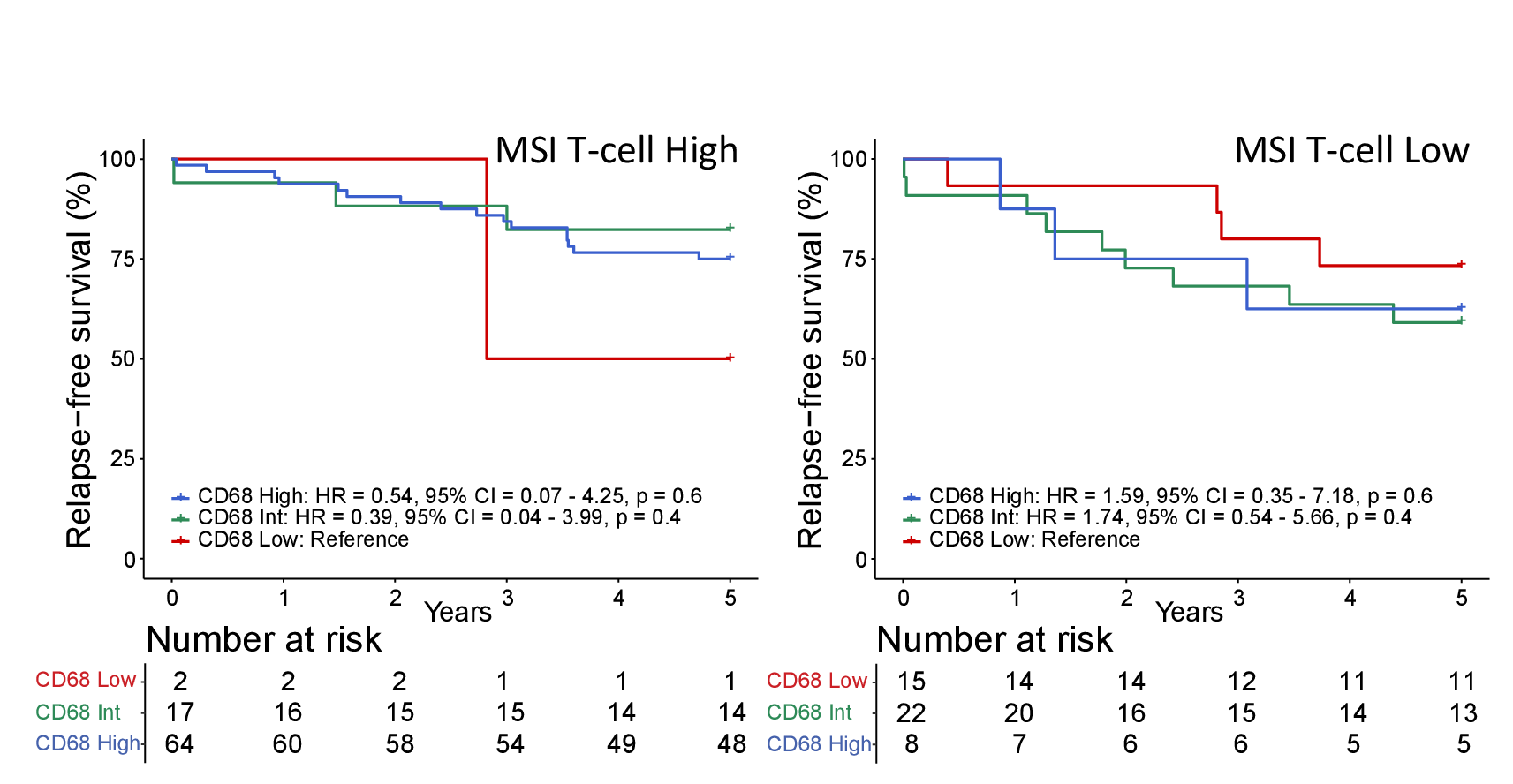

Supplement: Supplementary file 8 — Supplementary Material 8 [file 13402_2024_926_MOESM8_ESM.tiff]
