# Supplementary material for: The prognostic effect of tumor-associated macrophages in stage I-III colorectal cancer depends on T cell infiltration
Source: Cell Oncol (Dordr). 2024 Feb 26;47(4):1267–76. doi: 10.1007/s13402-024-00926-w (PMC11322253; doi:10.1007/s13402-024-00926-w)
Supplement: Supplementary file 9 — Supplementary Material 9 [file 13402_2024_926_MOESM9_ESM.pdf]

Supplemental Table 1

Total patients, n		Norwegian series 1 (1993-2003)	Norwegian series 2 (2003-2012)	Total
		922	798	1720
**Age**	Median (range)	73 (29–94)	72 (27–97)	72 (27 – 97)
**Sex**	Female	485 (53%)	407 (51%)	892 (52%)
	Male	437 (47%)	391 (49%)	828 (48%)
**TNM stage**	I	137 (15%)	167 (21%)	304 (18%)
	II	381 (41%)	288 (36%)	669 (39%)
	III	242 (26%)	214 (27%)	456 (27%)
	IV	159 (17%)	129 (16%)	288 (16)
	NA	3	-	3
**pT – Tumor invasion**	1	37 (4%)	40 (5%)	77 (4%)
	2	127 (14%)	165 (21%)	292 (17%)
	3	662 (72%)	515 (65%)	1,177 (69%)
	4	96 (10%)	72 (9%)	168 (10%)
	NA	0	6	6
**pN – Nodal Involvement**	0	563 (62%)	491 (62%)	1,054 (62%)
	1	250 (27%)	175 (22%)	425 (25%)
	2	99 (11%)	129 (16%)	228 (13%)
	NA	10	3	13
**Residual tumor (R) status**	R0	719 (78%)	651 (82%)	1,370 (80%)
	R1	36 (4%)	19 (2%)	55 (3%)
	R2	167 (18%)	128 (16%)	295 (17%)
**Tumor location**	Right colon	365 (40%)	327 (41%)	692 (40%)
	Left colon	301 (33%)	239 (30%)	540 (31%)
	Rectum	231 (25%)	218 (27%)	449 (26%)
	Synchronous	25 (3%)	14 (2%)	39 (2%)
**MSI status**	MSI	128 (14%)	120 (16%)	248 (16%)
	MSS	712 (85%)	638 (84%)	1,350 (84%)
	NA	82	40	122
***BRAF*^{V600E} mutational status**	Wild-type	714 (85%)	637 (84%)	1,351 (84%)
	Mutated	127 (14%)	122 (16%)	249 (16%)
	NA	81	39	120
***KRAS* mutational status**	Wild-type	463 (69%)	238 (69%)	701 (69%)
	Mutated	204 (31%)	106 (31%)	310 (31%)
	NA	255	454	709
**Preoperative radiotherapy**	No	903 (98%)	738 (92%)	1,641 (95%)
	Yes	19 (2%)	60 (8%)	79 (5%)
**Adjuvant chemotherapy**	No	806 (87%)	609 (79%)	1,415 (84%)
	Yes	116 (13%)	162 (21%)	278 (16%)
	NA	0	27	27
**Stromal CD68$^+$ per mm^2 (log2)**	Median (Q1-Q3)	8.8 (7.6 – 9.9)	8.7 (7.5 – 9.7)	8.8 (7.6 – 9.8)
**Stromal CD68$^+$CD163$^+$/CD68$^+$CD163$^-$ ratio**	Median (Q1-Q3)	0.78 (0.23 – 2)	0.74 (0.25 – 1.75)	0.75 (0.23 – 1.86)
**Stromal CD68$^+$CD163$^+$/CD68$^+$ ratio**	Median (Q1-Q3)	0.45 (0.19 – 0.69)	0.43 (0.2 – 0.64)	0.44 (0.20 – 0.67)

Supplemental Table 2:

Staining procedure

**Cycle**	**PT-link buffer solution (deparaffinization/antigen retrieval/antibody stripping)**	**Antibody (Dilution)**	**Fluorophore (Dilution)**
1	EnVision FLEX Target retrieval solution, Low pH (DAKO/Agilent)	Anti-CD206 (1:1200)	Opal 620 (1:100)
2	High-pH buffer (Akoya)	Anti-CD163 (1:1000)	Opal 520 (1:100)
3	High-pH buffer (Akoya)	Anti-CD68 (1:3000)	Opal 690 (1:100)
4	Low-pH buffer (Akoya)	Epithelial markers	Opal 570 (1:200)
5	Low-pH buffer (Akoya)	-	DAPI (1 drop/mL)

Supplemental Table 3:

Reagents used for multiplex IHC.

Reagent	Dilution used	Incubation time (min)	Vendor	Product #
Anti-CD206, clone E2L9N	1:1200	30	Cell Signaling Technology	91992
Anti-CD163, clone EPR14643	1:1000	30	Abcam	ab188571
Anti-CD68, clone KP1	1:3000	30	DAKO/Agilent	M081401-2
Anti-E-cadherin, clone 36	1:20000	30	BD-Biosciences	610182
Anti-Cytokeratin, Clone 11	1:4000	30	Abcam	ab7753
Anti-Cytokeratin, type I/II, Clone AE1/AE3	1:2000	30	Thermo Fisher Scientific	MA5-13156
DAPI	1 drop per mL	5	Akoya	In kit, NEL810001KT
EnVision FLEX Target retrieval solution, Low pH	1:50	20	DAKO/Agilent	K8005
Low-pH buffer	1:10	20	Akoya	In kit, NEL810001KT
High-pH buffer	1:10	20	Akoya	AR9001KT
Blocking buffer/primary antibody dilution solution	Undiluted/according to primary antibody dilution	10/30	Akoya	In kit, NEL810001KT
Secondary antibody (anti-rabbit/anti-mouse)	Undiluted	10	Akoya	In kit, NEL810001KT
Opal fluorophores and buffer	Varies, see supplementary table X	10	Akoya	In kit, NEL810001KT
Prolong Diamond Antifade Mountant	Undiluted	-	Thermo Fisher Scientific	P36970
Wash buffer	1:20	3 x 2 min between each step during staining	DAKO/Agilent	K8007

Supplemental Table 4:

Thresholds for positivity of the markers within the two series. If a cell had a mean (normalized counts, total weighting in Inform software) nuclear signal above the threshold it was scored as positive, otherwise it was scored as negative for the marker.

	Norwegian Series 1	Norwegian Series 2
CD68 (Opal 690)	2.168	3.5
CD163 (Opal 520)	0.269	0.6

Supplemental Table 5: REMARK checklist[1]

	Item to be reported	Page no.
**INTRODUCTION**		
1	State the marker examined, the study objectives, and any pre-specified hypotheses.	1+2
**MATERIALS AND METHODS**		
Patients		
2	Describe the characteristics (e.g., disease stage or co-morbidities) of the study patients, including their source and inclusion and exclusion criteria.	3+5,<br>Sup. Table 1
3	Describe treatments received and how chosen (e.g., randomized or rule-based).	3
Specimen characteristics		
4	Describe type of biological material used (including control samples) and methods of preservation and storage.	3
Assay methods		
5	Specify the assay method used and provide (or reference) a detailed protocol, including specific reagents or kits used, quality control procedures, reproducibility assessments, quantitation methods, and scoring and reporting protocols. Specify whether and how assays were performed blinded to the study endpoint.	3-5<br>Sup. Tables 2-4<br>Sup Fig. 1
Study design		
6	State the method of case selection, including whether prospective or retrospective and whether stratification or matching (e.g., by stage of disease or age) was used. Specify the time period from which cases were taken, the end of the follow-up period, and the median follow-up time.	3+1
7	Precisely define all clinical endpoints examined.	5
8	List all candidate variables initially examined or considered for inclusion in models.	3
9	Give rationale for sample size; if the study was designed to detect a specified effect size, give the target power and effect size.	3,6
Statistical analysis methods		
10	Specify all statistical methods, including details of any variable selection procedures and other modelbuilding issues, how model assumptions were verified, and how missing data were handled.	5-6
11	Clarify how marker values were handled in the analyses; if relevant, describe methods used for cutpoint determination.	5-6
**RESULTS**		
Data		
12	Describe the flow of patients through the study, including the number of patients included in each stage of the analysis (a diagram may be helpful) and reasons for dropout. Specifically, both overall and for each subgroup extensively examined report the numbers of patients and the number of events.	6<br>Sup Fig. 1
13	Report distributions of basic demographic characteristics (at least age and sex), standard (disease-specific) prognostic variables, and tumor marker, including numbers of missing values.	S. Table 1<br>Table 1
Analysis and presentation		
14	Show the relation of the marker to standard prognostic variables.	6-7<br>Figure 1-3,
15	Present univariable analyses showing the relation between the marker and outcome, with the estimated effect (e.g., hazard ratio and survival probability). Preferably provide similar analyses for all other variables being analyzed. For the effect of a tumor marker on a time-to-event outcome, a Kaplan-Meier plot is recommended.	Table 1<br>Figure 1-3
16	For key multivariable analyses, report estimated effects (e.g., hazard ratio) with confidence intervals for the marker and, at least for the final model, all other variables in the model.	Table 1
17	Among reported results, provide estimated effects with confidence intervals from an analysis in which the marker and standard prognostic variables are included, regardless of their statistical significance.	Figure 1-3,<br>Sup Figure 2-5
18	If done, report results of further investigations, such as checking assumptions, sensitivity analyses, and internal validation.	3-4, Sup Table 1, also reported in each table, if relevant.
**DISCUSSION**		
19	Interpret the results in the context of the pre-specified hypotheses and other relevant studies; include a discussion of limitations of the study.	7-8
20	Discuss implications for future research and clinical value.	7-8

1. McShane LM, Altman DG, Sauerbrei W, Taube SE, Gion M, Clark GM, et al. Reporting recommendations for tumor marker prognostic studies (REMARK). *J Natl Cancer Inst* **97**, 1180–4 (2005).
