# Supplementary material for: The prognostic effect of tumor-associated macrophages in stage I-III colorectal cancer depends on T cell infiltration
Source: Cell Oncol (Dordr). 2024 Feb 26;47(4):1267–76. doi: 10.1007/s13402-024-00926-w (PMC11322253; doi:10.1007/s13402-024-00926-w)
Supplement: Supplementary file 10 — Supplementary Material 10 [file 13402_2024_926_MOESM10_ESM.docx]

**Supplemental Figure captions**

A PDF file containing Supplemental Figures 1 to 8.

**Supplemental Fig. 1 Digital image analysis and marker scoring pipeline**

Flow-chart showing the steps in the digital image analysis and marker scoring pipeline.

**Supplemental Fig.2 Density of CD68^+^ TAMs according to cancer stage**

Comparison of TAM densities (stromal CD68^+^ per mm^2^, log2 transformed) according to stage of CRC. The p-value was estimated by the Kruskal-Wallis test. *Significant value P<0.05.

**Supplemental Fig.3 Density of CD68^+^ TAMs according to cancer stage and CD163 staining**

Comparisons of CD68^+^, CD68^+^CD163^-^ and CD68^+^CD163^+^ TAM densities between locoregional (stage I-III) and metastatic tumors (stage IV). P-values estimated by Wilcoxon test. *Significant values P<0.05.

**Supplemental Fig.4 Prognostic effect of CD68^+^ TAMs in stage I-III CRC split by patient cohort**

Kaplan-Meier plots of 5-year RFS according to the stromal density of CD68^+^ TAMs (high, intermediate, low) in the two patient cohorts (NS1 and NS2) analyzed separately. *Significant values P<0.05.

**Supplemental Fig.5 No prognostic value of CD68^+^ TAMs in stage IV CRC**

Kaplan-Meier plot of 5-year overall survival according to the stromal density of CD68^+^ TAMs (high, intermediate, low) among patients with stage IV CRC in the two patient cohorts combined.

**Supplemental Fig.6 Density of CD68^+^ TAMs relative to tumoral T cells in CRCs**

Correlation analysis of TAMs with stromal CD3 T cells (left) and epithelial CD8 T cells (right).

**Supplemental Fig.7 Survival analysis of CD68^+^ TAMs in CRCs with mixed levels CD3^+^ and CD8^+^ T cells**

Kaplan-Meier plot of 5-year RFS in patients with tumors with stromalCD3^high^epithelialCD8^low^ and stromalCD3^low^epithelialCD8^high^, stratified by the densities of TAMs.

**Supplemental Fig.9 Survival analysis of CD68^+^ TAMs according to T-cell densities in MSI tumors**

Kaplan-Meier plot of 5-year RFS in patients with T-cell^high^ and T-cell^low^ MSI tumors, stratified by the densities of TAMs.

**Supplemental Table captions**

A PDF file containing Supplemental Tables 1 to 5.

**Supplemental Table 1**

Molecular and clinicopathological characteristics of the two CRC series (NS1 and NS2).

**Supplemental Table 2**

Staining procedure for multiplex fluorescence immunohistochemistry.

**Supplemental Table 3**

Reagents used for multiplex fluorescence immunohistochemistry.

**Supplemental Table 4**

Thresholds for positivity of the markers within the two series.

**Supplemental Table 5**

REMARK checklist.
